# Akute Bauchschmerzen mit überraschender Ursache – eine seltene Komplikation einer häufigen Erkrankung

**DOI:** 10.1007/s00108-024-01804-1

**Published:** 2024-10-24

**Authors:** Katharina Grotemeyer, Roxana Motronea, Jörn M. Schattenberg

**Affiliations:** Klinik für Innere Medizin II, Universitätsklinikum des Saarlandes, Kirrberger Str. 100, 66424 Homburg/Saar, Deutschland

**Keywords:** Cholezystokolische Fistel, Akute abdominelle Schmerzen, Cholecystocolic fistula, Acute abdominal pain

## Abstract

Die Ausbildung einer cholezystokolischen Fistel als Komplikation der chronischen Cholezystitis und/oder Cholangitis ist sehr selten und lässt sich ohne moderne Bildgebung kaum nachweisen. Dennoch sind akute abdominelle Beschwerden eine häufige Ursache einer Vorstellung von Patienten in einer stationären Notaufnahme. Der vorliegende Fall demonstriert, dass auch ohne Vorliegen einer Cholestase und Infektkonstellation eine chronische Cholezystitis mit entsprechenden Komplikationen vorliegen kann.

## Abstract (en)

The development of a cholecystocolic fistula as a complication of chronic cholecystitis and/or cholangitis is very rare and is difficult to detect without modern imaging. Nevertheless, acute abdominal symptoms are a frequent cause of patient presentation to an emergency department. The present case demonstrates that chronic cholecystitis with corresponding complications can present even without cholestasis and infection.

## Anamnese

Ein 82-jähriger Patient stellte sich notfallmäßig mit seit zwei Tagen bestehenden diffusen abdominellen Schmerzen mit p. m. im Unterbauch sowie konsekutiver Übelkeit und Erbrechen an unserer interdisziplinären Notaufnahme vor. Fieber und/oder klinische Hinweise auf einen Infekt wurden verneint. Bekannt sind multiple abdominelle Voroperationen und eine chronische Obstipation. Die zuletzt durchgeführte Koloskopie lag 4–5 Jahre zurück und war anamnestisch unauffällig.

## Untersuchung

In der klinischen Untersuchung war das Abdomen weich bei jedoch diffusem Druckschmerz in allen vier Quadranten. Die Peristaltik war unauffällig und regelgerecht.

## Diagnostik

**Abb. 1:**
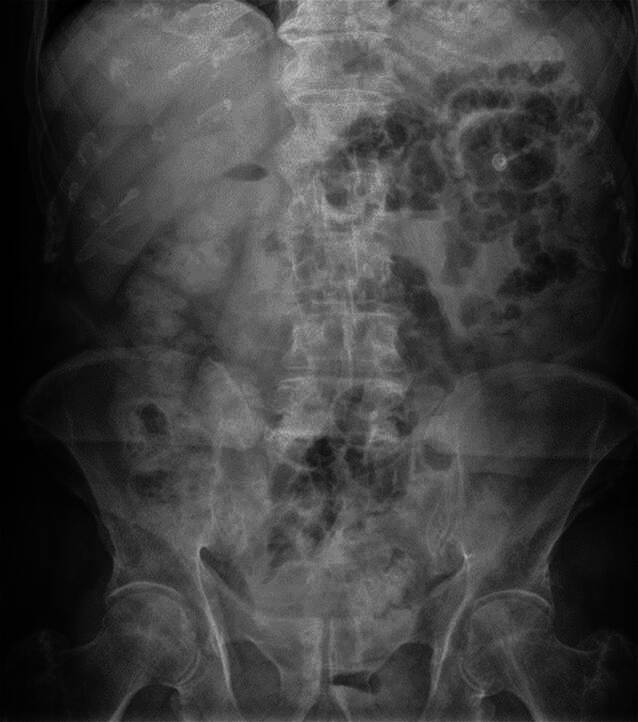
Abdomen Übersichtsaufnahme

Laborchemisch zeigten sich unauffällige Leberfunktionstests, das CRP sowie auch die Leukozyten lagen im Normbereich. Nebenbefundlich waren die Retentionsparameter leicht erhöht. In der initial angefertigten Abdomenübersichtsaufnahme (Abb. 1) zeigten sich mehrere gasgefüllte, nicht dilatierte Darmschlingen im linken Oberbauch sowie im Mittelbauch, ebenso ein stuhlgefülltes Colon ascendens im Rahmen der Koprostase. Hinweise auf Vorliegen eines Ileus ergaben sich nicht.

**Abb. 2:**
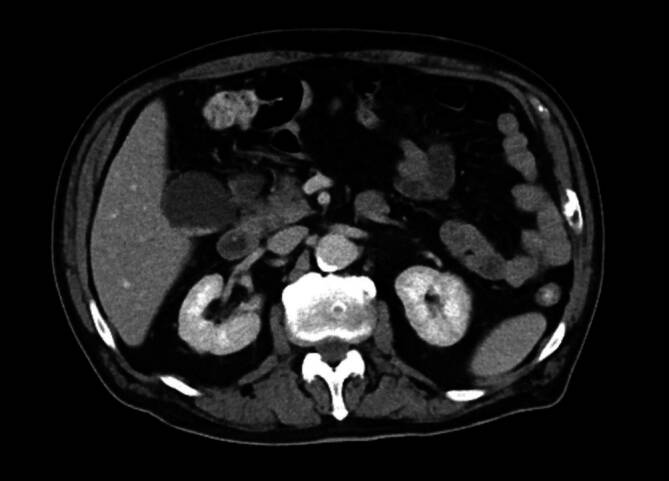
Computertomografie (CT) Abdomen mit Aerobilie und V.a. Vorliegen einer cholezystokolischen Fistel im Bereich des rechten Kolons

**Abb. 3:**
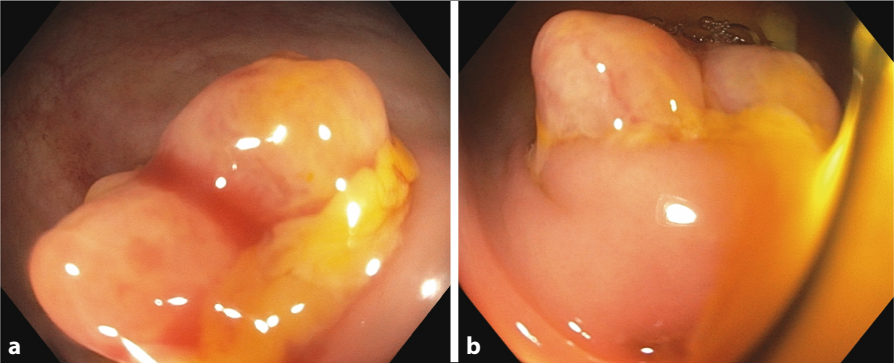
**a**, **b** Endoskopische Darstellung der cholezystokolischen Fistel mit papillenartiger Formation im Bereich des rechten Kolons mit Austritt von Gallenflüssigkeit

Bei persistierenden Beschwerden erfolgte im kurzfristigen Verlauf eine Computertomografie des Abdomens. Hier zeigten sich eine Aerobilie und eine fragliche Verbindung der Gallenblase zum rechten Kolon. Dieser radiologische Befund führte zum Verdacht auf eine Gallenfistel zwischen Gallenblase und rechter Kolonflexur (Abb. 2). In der Koloskopie zeigte sich im Bereich der rechten Flexur eine Schleimhautläsion bzw. Prominenz, die optisch einer Papille ähnelte und die Mündung des Fistelgangs darstellte. Es entleerte sich spontan gallige Flüssigkeit (Abb. 3).

## Therapie und Verlauf

Es erfolgte eine chirurgische Versorgung. Intraoperativ zeigte sich vernarbtes Gewebe zwischen dem Kolon und einer nicht sicher identifizierbaren Gallenblase bzw. dem Gallengang. Im Rahmen der Präparation konnte ein länglicher verhärteter Gewebsstrang zwischen diesen Strukturen isoliert werden, der a.e. der regressiv veränderten Gallenblase bzw. einem Fistelgang entsprach. Freie Gallenflüssigkeit im Bauchraum im Zusammenhang mit dieser Fistelung wurde nicht beobachtet. Es erfolgte letztendlich eine komplikationslose Cholezystektomie und Resektion des Fistelgangs mit einer Kolonresektion in der Größe eines 10-Cent-Stücks im Bereich der Fistelung.

In der pathologischen Begutachtung ergab sich das Bild einer chronisch-rezidivierenden Cholezystitis und Cholangitis (Ductus cysticus) mit chronischer Pericholezystitis sowie einem miterfassten reaktiven Hiluslymphknoten sowie Darmwand mit herdförmiger chronisch-granulierender, ulzeröser und hämorrhagischer Entzündung mit perifokaler Fibrose, welche der klinisch beobachteten Gallenblasen-Kolon-Fistel entsprach.

## Diskussion

Akute abdominelle Beschwerden sind eine häufige Ursache einer Vorstellung von Patienten in einer stationären Notaufnahme. Der vorliegende Fall demonstriert, dass auch ohne Vorliegen einer Cholestase eine chronische Cholezystitis mit entsprechenden Komplikationen vorliegen kann. Im Zweifel sollte bei persistierenden Beschwerden auch ohne Infektkonstellation eine bildgebende Diagnostik angestrebt werden.

Die Ausbildung einer cholezystokolischen Fistel als Komplikation der chronischen Cholezystitis und/oder Cholangitis ist sehr selten und lässt sich ohne moderne Bildgebung kaum nachweisen [1]. Häufiger sind solche Fistelungen bei malignen Erkrankungen der Gallenblase beschrieben [2]. Dies war jedoch bei dem beschriebenen Patienten nicht der Fall. Die endoskopische Untersuchung mit Auffinden der Fistelmündung unterstreicht, dass eine multimodale Diagnostik beim Erkennen seltener Erkrankungen unterstützen kann.
